# High-Throughput Virome Surveillance Reveals Emerging Viral Threats to Cucumber Production in Taiwan

**DOI:** 10.3390/v18101150

**Published:** 2026-10-16

**Authors:** Tsung-Chi Chen, Chian-Chi Lin, Po-Chen Chen

**Affiliations:** 1 Department of Medical Laboratory Science and Biotechnology, Asia University, Wufeng, Taichung 41354, Taiwan; 2 Doctoral Program in Microbial Genomics, National Chung Hsing University, Taichung 402202, and Academia Sinica, Taipei 115201, Taiwan

**Keywords:** *Cucumis sativus*, high-throughput sequencing, virome, emerging viruses, virus surveillance

## Abstract

Cucumber (*Cucumis sativus* L.) production in Taiwan is increasingly threatened by insect-transmitted viral diseases and complex mixed infections. In this study, symptomatic cucumber plants from a commercial net-house production system in Xizhou Township, Changhua County, Taiwan, were investigated using Illumina-based high-throughput sequencing (HTS) and virus-specific reverse transcription–polymerase chain reaction. A virome analysis conducted in 2022 identified five previously detected viruses, namely cucumber mosaic virus, cucumber Bulgarian latent virus, cucurbit chlorotic yellows virus (CCYV), melon yellow spot virus (MYSV), and watermelon silver mottle virus (WSMoV), and two emerging viruses, including a known virus, cucurbit chlorotic virus (CuCV), and an uncharacterized virus, tentatively designated cucumber tymovirus (CTyV). The complete genome of CuCV isolate XZ-C-1 was determined, and its sequence was closely related to previously reported CuCV isolates. A 5394-nt sequence of CTyV was also obtained and showed substantial divergence from its closest known viral relative. Importantly, comparative virus detection revealed a marked increase in the detection rates of several viruses between 2022 (*n* = 34) and 2025 (*n* = 30). MYSV remained highly prevalent (100.0% to 90.0%), whereas WSMoV increased dramatically from 2.9% to 96.7% and CCYV from 11.8% to 83.3%. The detection rates of CuCV and CTyV also increased from 20.6% to 53.3% and from 8.8% to 40.0%, respectively. In addition, all samples collected in 2025 harbored two to five viruses, indicating extensive mixed infections. These results reveal a dynamic and increasingly complex viral community in Taiwanese cucumber production systems and highlight the importance of continuous virome-based surveillance for early detection of emerging viruses and development of integrated disease-management strategies.

## 1. Introduction

Cucumber (*Cucumis sativus* L.) is one of the most important vegetable crops worldwide and represents a valuable component of protected vegetable production systems. Global cucumber production reached approximately 94.72 million tons in 2022, while Taiwan produced 47,083 tons from 1955 ha, ranking 57th worldwide [1]. Benefiting from its favorable climate, Taiwan supports year-round cucumber cultivation, particularly in central and southern regions. Pingtung County accounts for nearly half of the national production, followed by Kaohsiung, Yunlin, and Changhua [2]. As a high-value vegetable crop, cucumber contributes more than US$30 million annually to Taiwan’s agricultural economy [3].

Insect pests and viral diseases frequently threaten the productivity and profitability of cucumber cultivation, the latter being particularly destructive because effective control measures are limited. In addition to causing direct feeding damage, insect vectors such as aphids, thrips, and whiteflies facilitate rapid virus dissemination within and between production systems. Consequently, viral epidemics can lead to substantial reductions in yield, fruit quality, and marketability, resulting in significant economic losses for growers.

More than 16 viruses infecting cucurbit crops have been reported in Taiwan. Major cucurbit viruses include the aphid-transmitted cucumber mosaic virus (CMV), papaya ringspot virus watermelon type (PRSV-W), and zucchini yellow mosaic virus (ZYMV); the thrips-transmitted melon yellow spot virus (MYSV) and watermelon silver mottle virus (WSMoV); and the whitefly-transmitted cucurbit chlorotic yellows virus (CCYV) and squash leaf curl Philippines virus (SLCuPV) [4,5,6,7]. Despite the widespread use of insect-proof netting and other protected cultivation practices, insect-transmitted viral diseases remain a major challenge in cucumber production systems. In particular, MYSV and CCYV are among the most prevalent and economically damaging viruses affecting greenhouse-grown cucumbers in Taiwan [6].

Effective disease management depends on accurate and timely pathogen diagnosis. However, cucurbit crops in commercial production fields are frequently infected by multiple viruses simultaneously, often resulting in complex symptom expression and making disease diagnosis difficult. Conventional diagnostic methods, including enzyme-linked immunosorbent assay (ELISA) and reverse transcription–polymerase chain reaction (RT-PCR), are valuable tools for detecting known viruses but are less effective for identifying novel, divergent, or unexpected pathogens. As global trade, climate change, and vector population dynamics continue to influence the emergence and spread of plant viruses. The need for comprehensive virus surveillance has become increasingly important for sustainable crop production.

Recent advances in high-throughput sequencing (HTS) technologies have provided powerful tools for the comprehensive characterization of the plant virome. Illumina and Oxford Nanopore Technologies (ONT) are currently the most widely used sequencing platforms, offering complementary advantages in sequencing accuracy, read length, and field applicability [8]. HTS-based virome analysis enables the simultaneous detection of multiple viruses without prior knowledge of their presence and has substantially accelerated the discovery of emerging viruses in cucurbit crops. Examples include cucurbit vein banding virus [9], cucurbit cytorhabdovirus 1 [10], Chieh-qua chlorotic virus [synonym: cucurbit chlorotic virus (CuCV)], and chieh-qua endornavirus [11]. Beyond virus discovery, HTS has become an important tool for crop health monitoring, disease surveillance, germplasm and seed health testing, quarantine inspection, and the development of disease management strategies and virus-resistant cultivars [12].

In this study, severe viral disease outbreaks occurring in protected cucumber production facilities in Xizhou Township, Changhua County, central Taiwan, were investigated using Illumina-based HTS. Comprehensive virome analysis was conducted to characterize the viral communities associated with symptomatic cucumber plants and to identify potential emerging viral pathogens. In addition to detecting several previously detected cucurbit viruses, we identified emerging viruses that had not previously been recognized in cucumber production systems of Taiwan. The results provide valuable information on the diversity and composition of cucumber-associated viral communities, demonstrating that virome-based surveillance offers crucial insights for improving disease diagnosis and integrated pest management in cucumber production.

## 2. Materials and Methods

### 2.1. Sample Collection

**Figure 1:**
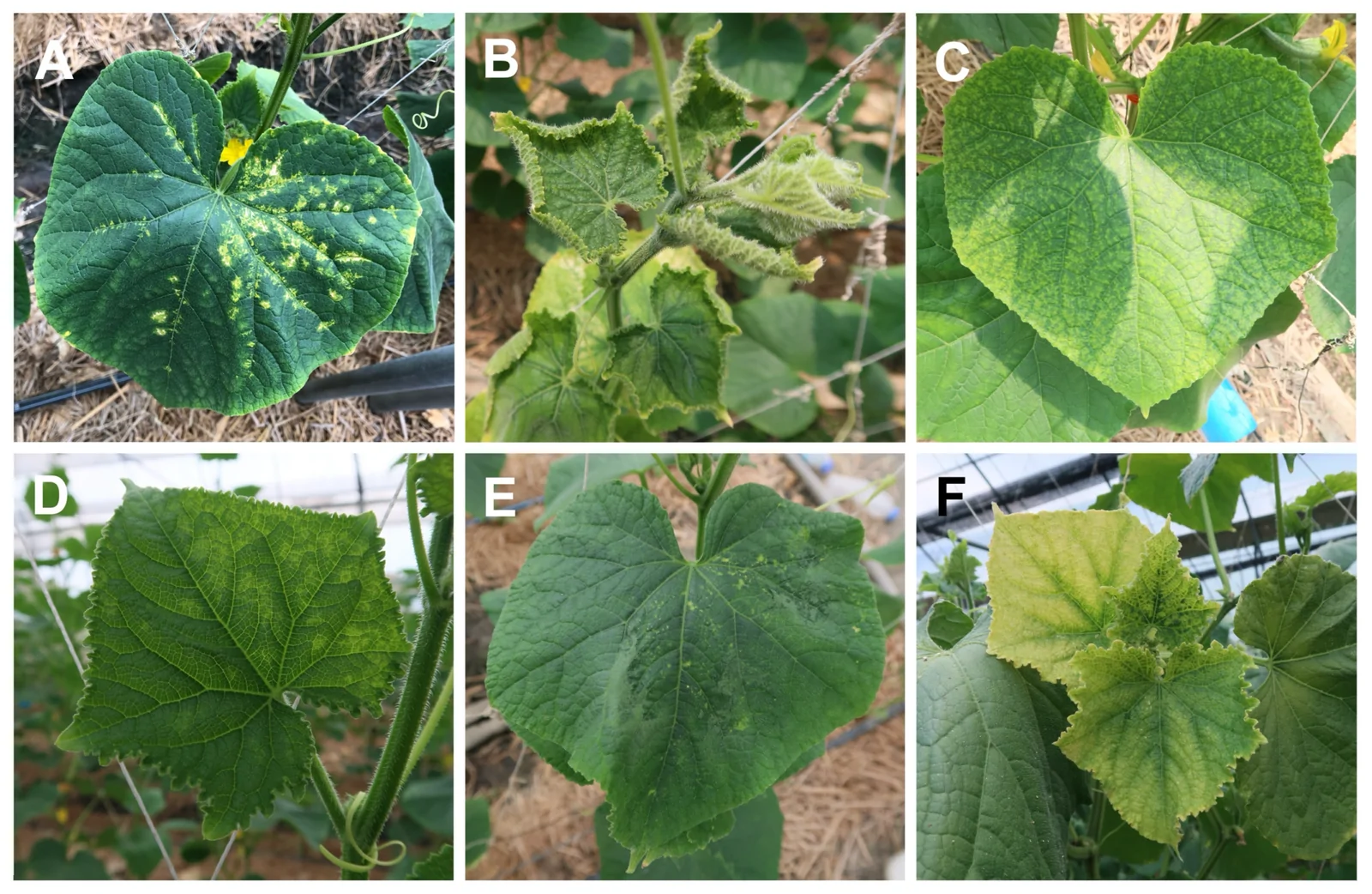
Symptoms observed in cucumber plants cultivated in commercial net-house production systems in Xizhou Township, Changhua County, Taiwan. (**A**) Yellow spotting associated with infection by melon yellow spot virus (MYSV). (**B**) Upper leaf curling associated with MYSV infection. (**C**) Mosaic associated with co-infection by MYSV and watermelon silver mottle virus (WSMoV). (**D**) Chlorotic spotting associated with co-infection by MYSV and cucumber mosaic virus (CMV). (**E**) Yellow spotting and mottling associated with co-infection by MYSV, cucurbit chlorotic virus (CuCV), and cucumber tymovirus (CTyV). (**F**) Upper leaf yellowing associated with co-infection by MYSV, CuCV, cucurbit yellows virus (CCYV), and cucumber Bulgarian latent virus (CBLV).

Two batches of symptomatic cucumber samples (CU-223, Ho Sheng Seeds, Tainan, Taiwan) (Figure 1) were collected from commercial net-house production systems in Xizhou Township, Changhua County, Taiwan. Batch 1 consisted of 34 leaf samples collected on 10 August 2022, whereas Batch 2 comprised 27 leaf samples and 3 fruit samples collected on 10 December 2025.

### 2.2. Total RNA Extraction

Total RNA was extracted from cucumber samples using the Total RNA Miniprep Purification kit (GMbiolab, Taichung, Taiwan) according to the manufacturer’s instructions. The purified RNA was stored at −80 °C until further use.

### 2.3. HTS Analysis

Equal amounts of total RNA (10 ng per sample) from the Batch 1 of 34 samples were mixed and then used to construct a random primer-based complementary DNA (cDNA) library using the Universal RNA-Seq Library Preparation kit with NuQuant (Tecan, Männedorf, Switzerland) according to the manufacturer’s instructions. The quantity and quality of the resulting cDNA library were assessed using a Qubit^®^ 2.0 Fluorometer with the dsDNA High Sensitivity kit (ThermoFisher Scientific, Waltham, MA, USA) and an Agilent 2100 Bioanalyzer (Agilent, Santa Clara, CA, USA). Paired-end sequencing and subsequent bioinformatics analyses were performed by Biotools Co., Ltd. (New Taipei City, Taiwan). Library sequencing was conducted on the NovaSeq 6000 platform (Illumina, San Diego, CA, USA). Ribosomal RNA reads were removed using the SILVA database [13]. The remaining reads were de novo assembled using Trinity software v2.4.0 [14]. Redundant sequences were clustered and removed using CD-HIT-EST v4.6 [15], generating a nonredundant set of contigs. The assembled contigs were subsequently subjected to BLASTn and BLASTx (BLAST+ v2.17.0) searches against the National Center for Biotechnology Information (NCBI) database for viral annotation and identification.

### 2.4. Genome Sequence Verification of Emerging Viruses

Total RNA extracted from cucumber samples that tested positive for emerging viruses was used as the template for viral genome verification. Primer pairs were designed based on assembled contigs and reference viral sequences (Supplementary Table S1) to amplify overlapping fragments spanning the complete viral genomes. First-strand cDNA synthesis was performed using SuperScript IV reverse transcriptase (SSIV RTase) (ThermoFisher Scientific, Waltham, MA, USA) with 2 μg of total RNA and 200 nM reverse primer at 50 °C for 10 min, followed by enzyme inactivation at 80 °C for 10 min. PCR amplification was carried out in a 25-μL reaction containing 2 μL of cDNA template, 1.25 U Blend Taq-Plus DNA polymerase (Toyobo, Osaka, Japan), 200 μM dNTPs, and 200 nM each of forward and reverse primers. The PCR program consisted of an initial denaturation at 94 °C for 3 min, followed by 35 cycles of 94 °C for 30 s, 58 °C for 1 min, and 72 °C for 3 min, with a final extension at 72 °C for 7 min. Amplified products were analyzed by electrophoresis on 1% agarose gels and eluted using the Micro-Elute DNA Clean/Extraction kit (GeneMark, Taichung, Taiwan) according to the manufacturer’s instructions. Eluted PCR products were cloned using the Invitrogen TOPO TA cloning kit (ThermoFisher Scientific, Waltham, MA, USA), ligated with the pCR2.1-TOPO vector. The ligation products were introduced into *E. coli* strain DH5α competent cells following the manufacturer’s protocol. Recombinant plasmids were purified from selected colonies and sequenced by Mission Biotech Co., Ltd. (Taipei, Taiwan) using an Applied Biosystems 3730XL DNA Analyzer (ThermoFisher Scientific, Waltham, MA, USA). Bidirectional Sanger sequencing was initially performed using M13 forward and reverse primers. Subsequently, sequence-specific primers were designed based on the obtained sequences to perform primer walking. For each amplicon, three independent clones were sequenced to ensure sequence accuracy.

### 2.5. Genome Sequence Analysis of Emerging Viruses

The genome sequences of the representative members of the genus *Crinivirus* (Supplementary Table S2) and the order *Tymovirales* (Supplementary Table S3) were retrieved from the NCBI GenBank database for comparative analyses. Pairwise nucleotide (nt) and amino acid (aa) sequence identities were calculated using Sequence Demarcation Tool Version 1.2 (SDTv1.2), which also generated color-coded identity matrices based on pairwise sequence alignments [16]. Multiple sequence alignments were performed using ClustalX version 2.1 [17]. Phylogenetic analyses were conducted using MEGA X v10.0.5 [18]. Maximum-likelihood (ML) phylogenetic trees were constructed based on the appropriate nt substitution models [19,20], and branch support was evaluated using 1000 bootstrap replicates. Viral protein functional domains were analyzed using InterPro v110.0 [21].

### 2.6. Virus Detection

Total RNA extracted from the collected cucumber samples was used as the template. The following Virus-specific primer pairs were used to detect corresponding viruses: CBLV3900F/CBLV4576R to CBLV [6], Crini-hsp70-f/Crini-hso70-r to CCYV [22], CMV-CP-Nco-F/CMV-CP-Sac-R to CMV (our laboratory, unpublished), MY-N516f/MY-N774r to MYSV [23], WS-N572f/WS-N808r to WSMoV [24], TMVmpf/TMV3nr to TMV [25], and CuCV-R2-1F/1R to CuCV (designed in this study). Primer pairs c40723-1F/c40723-1R, c43195-1F/c43195-1R, and c44091-1F/c44091-1R were used to detect unclassified viruses. Primer sequences are listed in Supplementary Table S4.

MYSV and WSMoV were tested by SYBR Green I-based real-time RT-PCR as described previously [23]. Testing was conducted using the QuantStudio 1 Real-Time PCR System (Applied Biosystems, Thermo Fisher Scientific, Waltham, MA, USA). The reaction mixture (10 µL) included 2 µL total RNA, 5 µL KAPA SYBR^®^ FAST qPCR 2 × Master mix (KAPA Biosystems Inc., Woburn, MA, USA), 50 U SAMscript reverse transcriptase (GMbiolab, Taichung, Taiwan), 10 U RNase inhibitor (GMbiolab, Taichung, Taiwan), and 100 nM of each primer. The reaction conditions were cDNA synthesis at 42 °C for 30 min, DNA polymerase activation at 95 °C for 5 min, followed by 35 cycles of 95 °C for 15 s, 65 °C for 30 s, and 75 °C for 20 s. Melting curve analysis was performed from 65 °C to 95 °C with a 0.3 °C increment. Reactions were considered positive if both an exponential increase in fluorescence and a specific melting peak were detected.

Other viruses were tested by conventional RT-PCR. Amplification was performed by VeritiPro Thermal Cycler (Applied Biosystems, Thermo Fisher Scientific, Foster City, CA, USA) using the One-step RT-PCR kit (GeneMark, Taichung, Taiwan) according to the manufacturer’s instructions. Each 25-µl reaction contained 2 µL of total RNA, 12.5 µL of 2× RT buffer (containing dNTPs, Mg2^+^ and enzyme stabilizer), 0.5 µL of enzyme mix (reverse transcriptase and *Taq* DNA polymerase), 0.5 µL each of forward and reverse primers (final concentration, 200 nM), 0.1 µL of RNase block, and nuclease-free water to the final volume. The RT-PCR program consisted of RT at 50 °C for 30 min and initial denaturation at 94 °C for 2 min, followed by 35 cycles of 94 °C for 30 s, 58 °C for 30 s, and 72 °C for 1 min, with a final extension at 72 °C for 7 min. Amplified products were analyzed by electrophoresis on 1% agarose gels. To verify virus identity, representative amplicons were randomly selected for Sanger sequencing. Sequencing was performed by Mission Biotech Co., Ltd. (Taipei, Taiwan) using an Applied Biosystems 3730XL DNA Analyzer (ThermoFisher Scientific, Waltham, MA, USA).

### 2.7. Statistical Analysis

The virus detection rates were compared between the sampling periods of 2022 and 2025 to evaluate the temporal changes in virus prevalence. Fisher’s exact test was employed to determine statistical significance for individual viruses [26]. To further characterize the precision of the estimated prevalence, 95% confidence intervals (CIs) for individual detection rates were calculated using the Wilson score method [27]. All statistical tests were two-tailed, and a *p*-value of less than 0.05 was considered statistically significant. Statistical analyses were performed using standard statistical formulas.

## 3. Results

### 3.1. Illumina Sequencing Data Analysis and Contig Assembly

**Figure 2:**
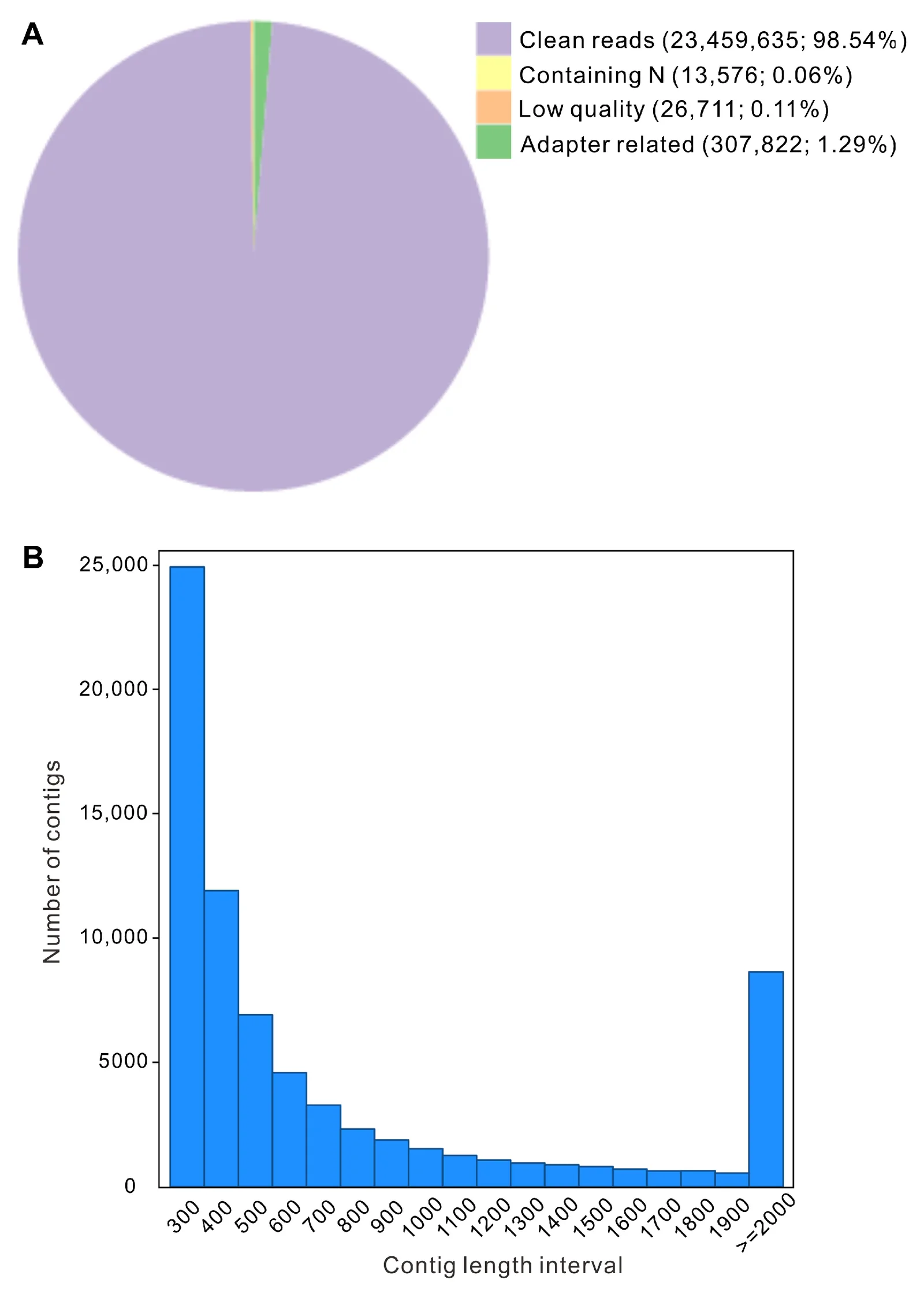
Analysis of reads generated from Illumina-based high-throughput sequencing. (**A**) Classification of raw reads. (**B**) Contig length distribution.

Illumina sequencing generated 23,807,744 raw reads from the total RNA mixture of the Batch 1 samples. After removal of adaptor sequences, ambiguous reads, and low-quality reads, 23,459,635 clean reads were retained for downstream analysis. De novo assembly of the clean reads yielded 73,518 contigs, with N50 and N90 values of 1708 and 341 bases, respectively (Table 1 and Figure 2).

### 3.2. Contig Annotation Against Viruses

The assembled contigs were subsequently subjected to BLAST searches against the NCBI database for viral annotation. A total of 22 contigs were annotated to nine viruses. Seventeen contigs showed 86.7–99.9% base identities to the genomes of six viruses, namely cucumber Bulgarian latent virus (CBLV), CCYV, CuCV, CMV, MYSV, and WSMoV, and cover almost their entire genome sequences. A short contig of 724 bases was closely related to tobacco mosaic virus (TMV), with 98.8% identity. Except for CuCV, all of these viruses had previously been detected in Taiwan. Additionally, four contigs were annotated to, but distinct from, two novel viruses: three to blackberry virus X (BVX), an unclassified virus of the order *Tymovirales*, and one to Sunn-hemp crinivirus (ShCV), an unclassified crinivirus. This suggested the possible existence of unknown viruses. The vial annotation results are summarized in Table 2.

### 3.3. Verification of the Presence of Emerging Viruses

**Figure 3:**
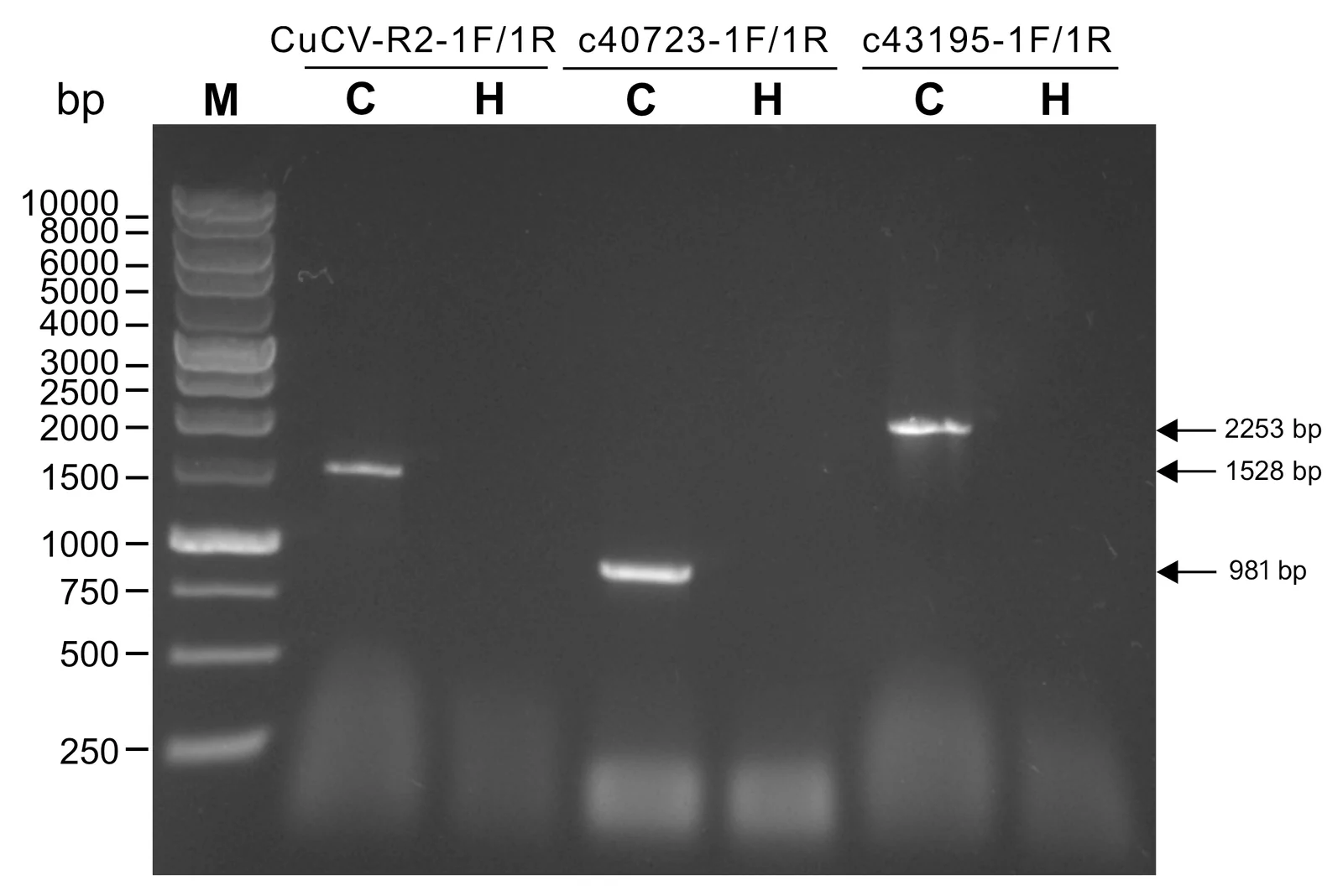
Verification of the presence of the emerging viruses by reverse transcription–polymerase chain reaction. The primer pair CuCV-R2-1F/1R was used to detect cucurbit chlorotic virus (CuCV). Primer pairs c40723-1F/1R and c43195-1F/1R were used to detect blackberry virus X-annotated unknown viruses. C represents the total RNA mixture of cucumber samples collected on August 10, 2022. H represents total RNA extracted from a healthy cucumber plant. The expected sizes of amplicons are indicated by arrows.

The total RNA mixture used for HTS was tested for the presence of annotated emerging viruses by RT-PCR. CuCV and unknown viruses annotated to BVX and ShCV were tested using primers designed based on the corresponding contig sequences (Supplementary Table S4). Because the BVX-annotated contigs c43195_g2 and c43195_g1 shared head-to-tail overlapping sequences, forward and reverse primers c41395-1F and c41395-1R were designed from contigs c43195_g2 and c43195_g1, respectively, to assess if the two contigs belong to the same virus. The results were positive for CuCV and the BVX-annotated unknown viruses (contigs c40723_g1, c43195_g2, and c43195_g1) (Figure 3), but negative for the ShCV-annotated unknown virus (contig c44091_g2). PCR products were sequenced to confirm their correctness. Additionally, the results also suggested that BVX-annotated contigs c40723_g1, c43195_g2, and c43195_g1 may be the same virus.

### 3.4. Clarification of the BVX-Annotated Unknown Virus

**Figure 4:**
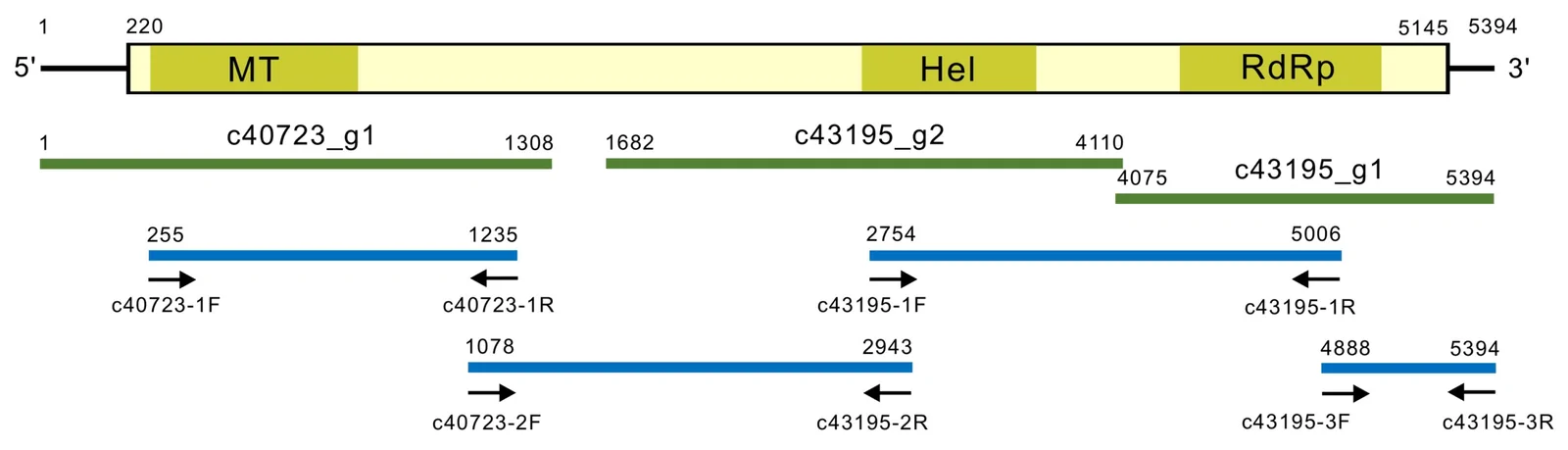
Schematic diagram for genome sequencing of the novel cucumber tymovirus (CTyV). Open reading frame (ORF) is represented by a yellow box, with the corresponding positions indicated by numbers. The predicted conserved domains, including to a putative methyltransferase (aa 42–343), helicase (aa 905–1139), and RNA-dependent RNA polymerase (aa 1375–1652), are indicated by dark yellow boxes within the ORF. Thick green lines represent the corresponding positions of contigs in the genome. Primers used for amplification of overlapping fragments (thick blue lines) are indicated by arrows.

Primers designed based on sequences of the three BVX-annotated contigs were used to successfully amplify four overlapping fragments covering the three contigs (Supplementary Table S1 and Figure 4). The overlapping fragments were cloned, and their sequences were determined by Sanger sequencing. After gap filling, a contiguous 5394-nt sequence was obtained, containing a single 1714-aa open reading frame (ORF). The predicted polyprotein contained conserved domains corresponding to a putative methyltransferase (MT, aa 42–343), helicase (Hel, aa 905–1139), and RNA-dependent RNA polymerase (RdRp, aa 1375–1652) (Figure 4).

**Figure 5:**
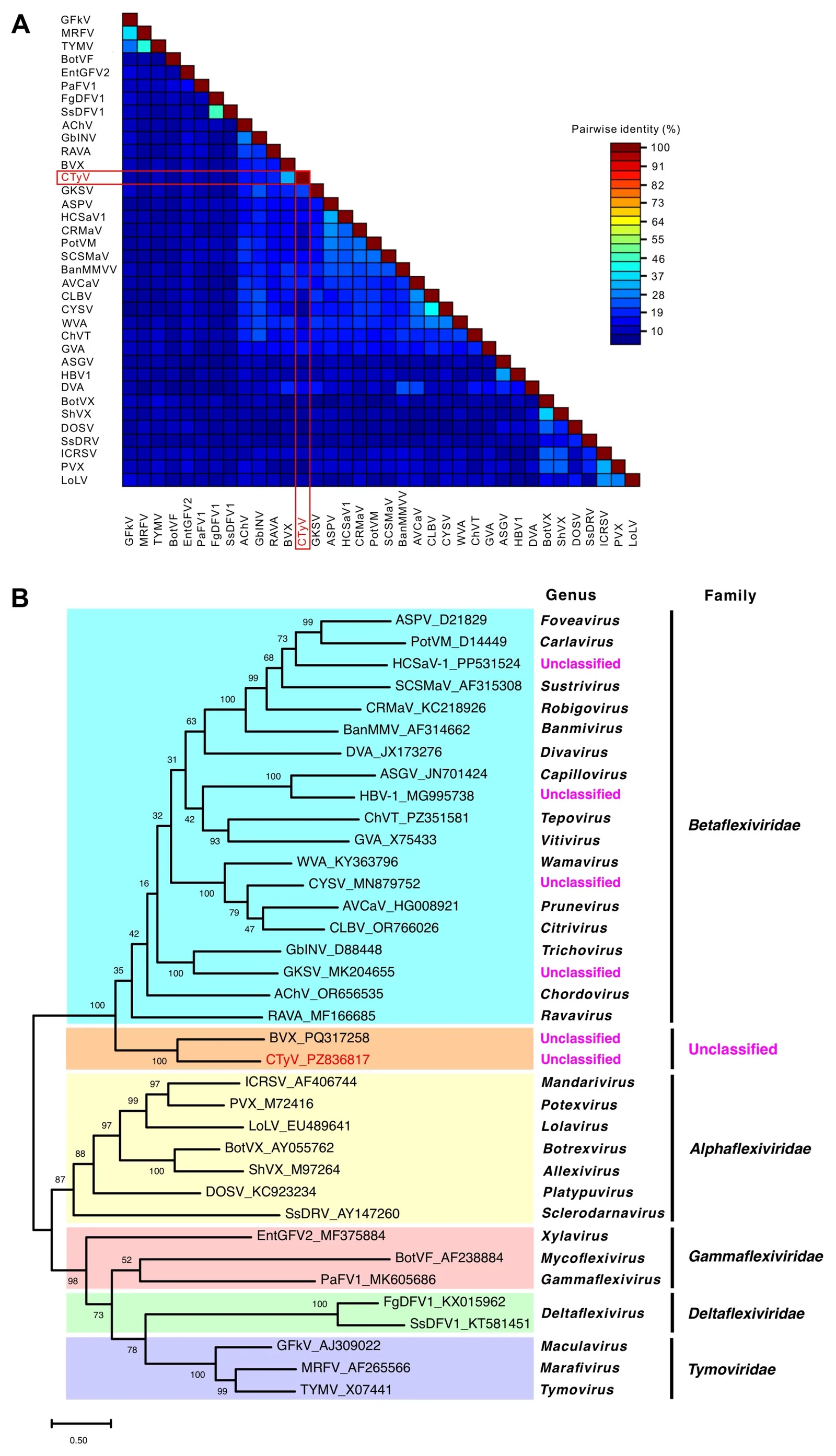
(**A**) Color-coded pairwise matrix and (**B**) phylogenetic analysis of the putative polyprotein encoded by cucurbit tymovirus (CTyV). Amino acid sequences of the replicase proteins from representative members of the order *Tymovirales* were included in both analyses. Pairwise Sequence Demarcation Tool Version 1.2 (SDTv1.2) was used to perform pairwise alignments and to generate the color-coded matrix. The phylogenetic tree was constructed using the maximum-likelihood method with 1000 bootstrap replicates in MEGA X v10.0.5, with evolutionary distances estimated using the maximum composite likelihood method. Representative viruses are labeled with their genus and family classifications. Viruses from different families are marked with boxes of different colors. CTyV is highlighted in red, and bootstrap values are indicated at the corresponding nodes.

BLAST searches against the NCBI database showed that the 5394-nt sequence shared the highest nt sequence similarity with BVX isolate IPW_116 (accession number PQ317260), with 67.3% nt identity over 12% of the query sequence. At the protein level, the predicted polyprotein shared 37.6% aa identity with 97% coverage of the BVX polyprotein. In the phylogenetic analysis, the uncharacterized virus and BVX formed a distinct lineage that was separated from the established virus groups included in the analysis, although this lineage showed an overall phylogenetic association with members of the order *Tymovirales*, including viruses currently classified in the family *Betaflexiviridae*, (Figure 5). Thus, the phylogenetic position of the uncharacterized virus suggests a relationship with betaflexivirus-like viruses but does not, on its own, provide sufficient evidence to assign it definitively to the family *Betaflexiviridae*.

Together, the sequence pairwise and phylogenetic analyses support the presence of a previously uncharacterized virus in the cucumber field, which we tentatively designated cucumber tymovirus (CTyV). However, because only a partial genome sequence was recovered and the available phylogenetic evidence places CTyV in a distinct lineage, its definitive taxonomic position within the order *Tymovirales* and its status as a distinct viral species cannot be established at this stage. Recovery and characterization of the complete genome, together with additional comparative and phylogenetic analyses, will be necessary for definitive taxonomic assignment according to the relevant ICTV criteria. The partial genome sequence of CTyV has been deposited in GenBank under accession number PZ836817.

### 3.5. Verification of CuCV Complete Genome Sequence

**Figure 6:**
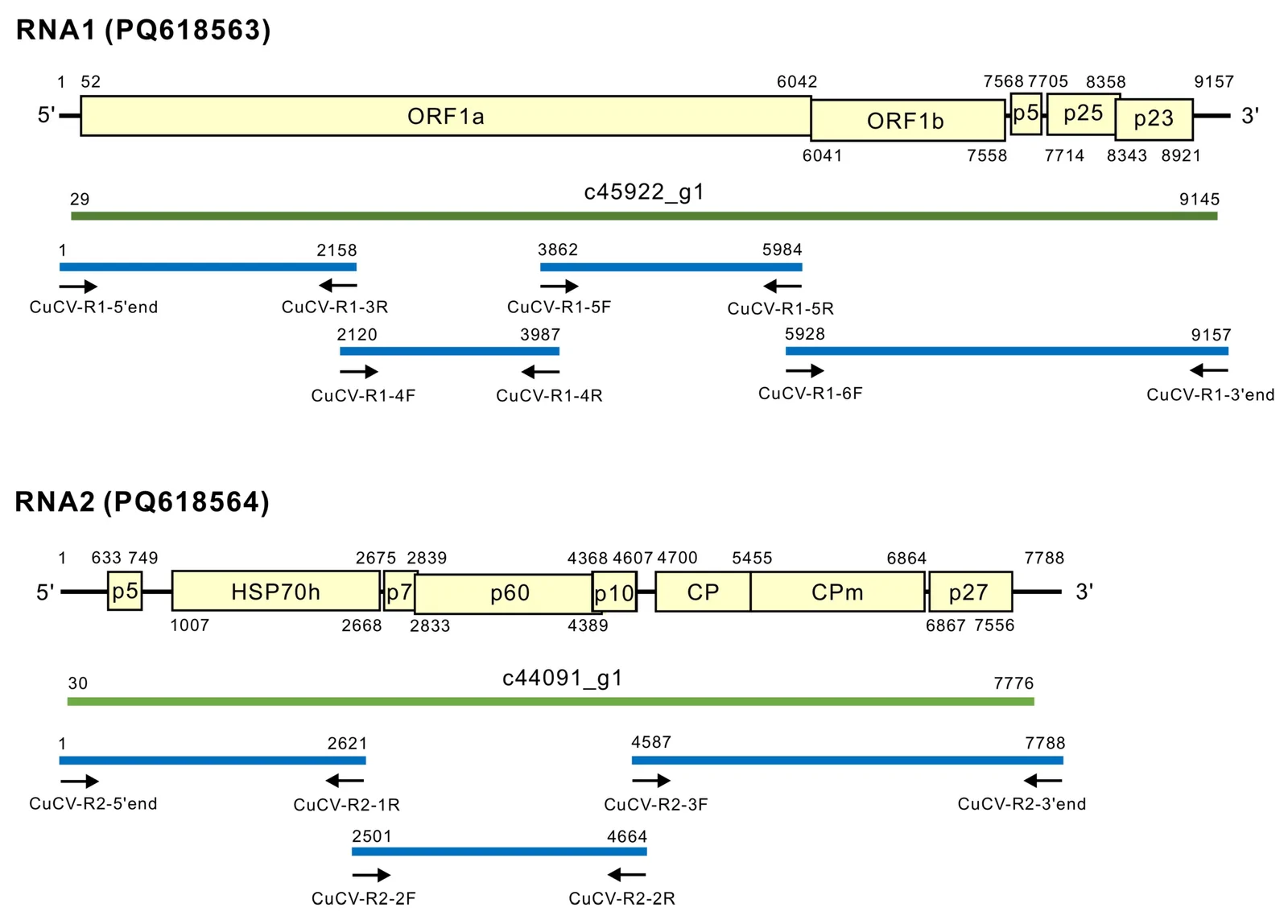
Schematic diagram for genome sequencing of cucurbit chlorotic virus (CuCV) isolate XZ-C-1. Open reading frames (ORFs) within RNA1 and RNA2 are represented by boxes, with their corresponding positions indicated by numbers. Thick green lines represent the corresponding positions of contig in the genome. Primers used for amplification of overlapping fragments (thick blue lines) are indicated by arrows. Accession numbers of RNA1 and RNA2 deposited in GenBank are shown.

Because CuCV was first detected in Taiwan, its complete genome sequence was also verified by Sanger sequencing. Primers used in RT-PCR amplification were designed based on the annotated contig sequences and the available CuCV genome sequences in the GenBank database (Supplementary Table S1). Complete RNA1 and RNA2 sequences were obtained from overlapping fragments, as illustrated in Figure 6. The RNA1 and RNA2 of the virus, denoted as XZ-C-1, were determined to be 9157 nt and 7788 nt long, respectively, a similar structure and length to those of characterized CuCV isolates. Their sequences are nearly identical to the contig c45922_g1 and c44091_g1 sequences, and have been deposited in GenBank with accession numbers PQ618563 and PQ618564.

**Figure 7:**
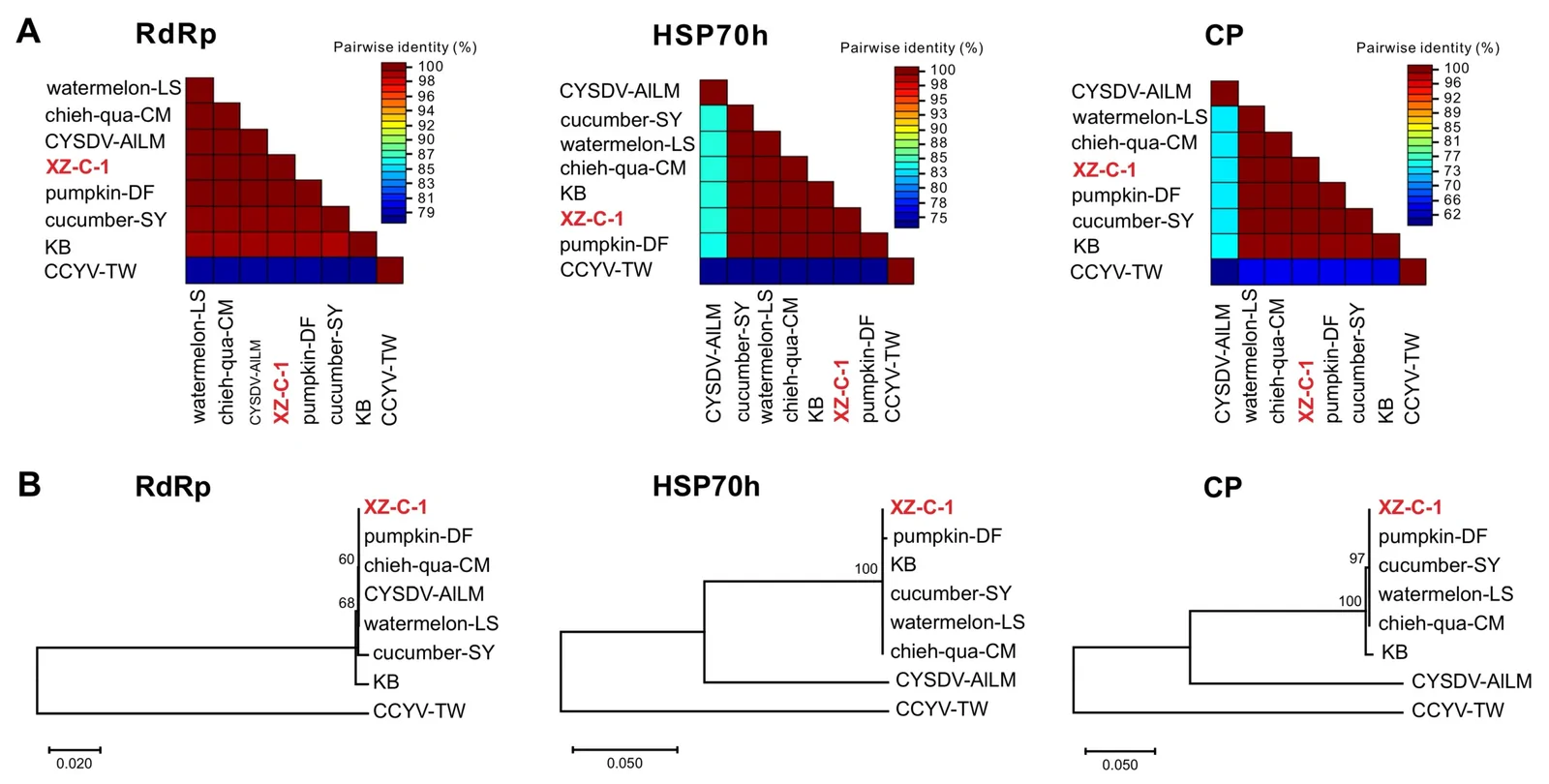
(**A**) Color-coded pairwise matrix and (**B**) phylogenetic analysis of key proteins encoded by cucurbit chlorotic virus (CuCV) XZ-C-1 isolate. Pairwise Sequence Demarcation Tool Version 1.2 (SDTv1.2) was used to perform pairwise alignments and to generate the color-coded matrix. The CuCV isolate XZ-C-1 determined in this study is highlighted in red. The cucurbit yellow stunting disorder virus (CYSDV) isolate AlLM and the Taiwanese cucurbit chlorotic yellows virus (CCYV) isolate TW were used as outgroups for comparison. The phylogenetic trees were constructed by the Maximum-likelihood method with 1000 bootstrap replicates using MEGA X v10.0.5, and the maximum composite likelihood method was used to compute evolutionary distances. The accession numbers of viral genome sequences are shown in Supplementary Table S2.

The full-length nt sequences of XZ-C-1 RNA1 and RNA2 were aligned with those of other CuCV isolates, sharing high identities of 98.3–99.8% and 99.3–99.9%, respectively. By comparison with the whole genome sequences of representative cucurbit yellow stunting disorder virus (CYSDV) and CCYV isolates, 59.7–66.8% nt identities were shown. The putative proteins encoded by RNA1 and RNA2 of XZ-C-1 were also closely related to those of other CuCV isolates, with 92.1–100.0% aa identities (Supplementary Table S5). The results of pairwise comparison and phylogenetic analysis showed that XZ-C-1 clustered with CuCV isolates, but was distant from CYSDV and CCYV, and was therefore a CuCV isolate (Figure 7).

### 3.6. Virus Occurrences in the Same Cucumber-Producing Area in 2022 and 2025

Cucumber samples from Batch 1 were individually tested for six previously detected viruses, CBLV, CMV, CCYV, MYSV, WSMoV, and TMV, and two emerging viruses, CuCV and CTyV. All 34 samples (100%) tested positive for MYSV, whereas none tested positive for TMV. Seven samples (20.6%) were positive for CuCV, four (11.8%) for CCYV, three (8.8%) each for CMV and CTyV, and one (2.9%) each for CBLV and WSMoV (Table 3). Fourteen samples (41.2%) were co-infected with two to four viruses. Overall, seven of the nine viruses targeted by the individual assays were detected in Batch 1.

Furthermore, a total of 30 symptomatic cucumber samples (Batch 2), including 27 leaf samples and 3 fruit samples, were collected on December 10, 2025, to investigate the occurrences of the nine annotated viruses in the same cucumber-producing area. The same method was used for virus testing. None of the samples were positive for CMV, TMV, and the ShCV-annotated hypothetical virus. A total of 29 samples (96.7%) were positive for WSMoV, followed by 27 (90.0%) for MYSV, 25 (83.3%) for CCYV, 16 (53.3%) for CuCV, 12 (40.0%) for CTyV, and five (16.7%) for CBLV (Table 3). All tested samples were co-infected with two to five viruses. In conclusion, six viruses were detected in this survey.

The prevalence of individual viruses was statistically compared between the two sampling periods to evaluate temporal changes in viral incidence. From 2022 to 2025, significant increases in the detection rates of several viruses were observed. These statistical results provide further evidence that the increased viral disease burden observed in 2025 was associated with significantly higher detection rates of WSMoV, CCYV, CuCV, and CTyV (Table 3).

## 4. Discussion

Viral diseases are an important constraint to cucumber production in Taiwan, particularly in protected cultivation systems where favorable environmental conditions and continuous cropping can facilitate virus and vector spread. In this study, Illumina-based HTS provided a comprehensive overview of viruses associated with symptomatic cucumber plants in a commercial net-house production area in Xizhou Township, Changhua County. The analysis identified six previously reported viruses, CBLV, CCYV, CuCV, CMV, MYSV, and WSMoV, as well as a previously unrecognized virus, tentatively designated cucumber tymovirus (CTyV). This finding demonstrates that the viral complexity of cucumber production systems may be greater than indicated by routine pathogen-specific diagnostics.

The identification and characterization of the emerging CuCV is of particular significance for cucumber production in Taiwan. The complete genome of the CuCV XZ-C-1 isolate consisted of RNA1 and RNA2 and showed 98.3–99.8% and 99.3–99.9% nt identity, respectively, with previously reported CuCV isolates. Phylogenetic analysis further placed XZ-C-1 within the CuCV cluster and clearly separated it from CCYV, another cucurbit-infecting crinivirus prevalent in Taiwan. These results confirm the occurrence of CuCV in the investigated cucumber production system and expand the known distribution of this virus in Taiwan. To our knowledge, CCYV and CuCV cause similar interveinal chlorosis and yellowing symptoms in the lower leaves of cucurbit hosts [11,28]. Previously, we have developed an RT-PCR method to detect CCYV in the field [22]. In this study, we developed a CuCV-specific RT-PCR detection method to distinguish between them. The host range and distribution of CuCV in Taiwan will be further investigated to elucidate its impacts on the agricultural system.

The discovery of CTyV provides further evidence that emerging viruses can remain undetected when surveillance relies exclusively on established diagnostic targets. BLASTn analysis showed that the assembled CTyV nt sequence shared 67.3% identity with BVX, an unclassified virus related to the family *Betaflexiviridae* within the order *Tymovirales* [29]; however, this nt identity was observed over only 12% of the CTyV sequence. Thus, the 67.3% identity does not represent genome-wide similarity between CTyV and BVX, but rather the similarity within a limited aligned region. This limited sequence similarity supports the distinction of CTyV from BVX while suggesting a relatively distant evolutionary relationship. At the protein level, the predicted CTyV polyprotein shared 37.6% aa identity with the BVX polyprotein across 97% of the BVX protein, and both proteins contained similar predicted functional domains, including putative MT, Hel, and RdRp domains [29]. The phylogenetic relationship between CTyV and BVX further supports an evolutionary association between the two viruses; however, their placement in a distinct lineage does not provide sufficient evidence for definitive family-level classification of CTyV.

The assembled 5394-nt sequence likely represents a partial genome of CTyV. Because the complete genome organization and terminal sequences remain unresolved, CTyV should currently be regarded as a tentative novel virus. Complete genome sequencing, including determination of the 5′ and 3′ terminal regions using rapid amplification of cDNA ends (RACE), will be necessary to fully characterize the genome and evaluate its taxonomic status according to the relevant ICTV criteria. Additionally, the repeated detection of CTyV in cucumber samples warrants further investigation of its host range, transmission biology, geographic distribution, and potential effects on cucumber production and crop performance.

The detection of multiple viruses in the same production system is particularly relevant to disease management. In 2022, 41.2% of the tested samples were co-infected, whereas all samples collected in 2025 were co-infected with two to four viruses. Mixed infections in a plant are common and can produce overlapping symptoms and complicate field diagnosis, making symptom-based identification unreliable. Similar mixed infections have been previously reported in cucurbit production systems in Taiwan [6,30] and emphasize the value of HTS as a complementary diagnostic approach for detecting multiple viruses simultaneously.

It should be noted, however, that pooling total RNA samples from Batch 1 into a single HTS library represents a methodological trade-off. While RNA pooling is a practical and cost-effective approach for preliminary virome screening and novel virus discovery across field populations, it inherently precludes the direct assessment of individual viral loads or specific co-infection frequencies within single host plants from the HTS data. To overcome this limitation, subsequent targeted assays, such as RT-PCR and ELISA, of individual plant samples were essential to establish exact incidence rates, determine co-infection spectra, and confirm the true field prevalence of both previously detected and newly identified viruses. Thus, HTS should be viewed not as a replacement for routine diagnostic assays, but as a powerful discovery and surveillance platform that can guide the development and application of specific RT-PCR assays for rapid monitoring of newly recognized viruses.

The high detection rates of MYSV, 100% in 2022 and 90.0% in 2025, as well as our previous report [6], indicate it is one of the most prevalent cucumber-infecting viruses in Taiwan. A notable finding was the statistically significant increase in the detection rates of several viruses between the two sampling periods. WSMoV increased from 2.9% to 96.7%, CCYV increased from 11.8% to 83.3%, CuCV increased from 20.6% to 53.3%, and CTyV increased from 8.8% to 40.0%. These marked increases indicate that these viruses were detected in a substantially greater proportion of cucumber samples in 2025 than in 2022. MYSV and WSMoV are primarily associated with transmission by *Thrips palmi* [31,32], whereas CCYV and CuCV are associated with transmission by *Bemisia tabaci* [33]. Both *T. palmi* and *B. tabaci* were readily observed in the net houses during the study periods, suggesting that vector pressure may have contributed to the high prevalence of their associated viruses. Nevertheless, because vector abundance and transmission efficiency were not quantitatively assessed, a direct relationship between vector populations and virus prevalence cannot be established from the present data. The high virus prevalence observed in 2025 nevertheless suggests substantial and persistent vector-mediated disease pressure within this protected production system.

In fact, two common aphid-transmitted cucurbit viruses, PRSV and ZYMV, were also tested in 2022 and 2025, but all cucumber samples were negative for them. This supports the HTS analysis results. CMV can also be associated with aphid transmission. The low detection rate of CMV, 8.8% in 2022, but no positives in 2025, is consistent with few aphids being found in the net houses.

The coexistence of viruses transmitted by different insect vectors has important implications for integrated disease management. Although insect-proof netting can reduce vector entry, it may not provide complete protection against small-bodied insects such as thrips and whiteflies, particularly when mesh specifications, structural integrity, or maintenance are inadequate. In addition, the relatively stable environmental conditions within net houses may favor the persistence and reproduction of insect vectors. Continuous or repeated cucumber cultivation may further provide a continuous host resource for both viruses and their vectors, potentially contributing to sustained disease pressure. Intensive insecticide use may also reduce control efficacy if applications are poorly timed or if vector populations develop resistance. These considerations highlight the need for integrated management combining effective vector exclusion, monitoring of vector populations, appropriate insecticide rotation and resistance management, removal of infected plants, and regular virus surveillance. Such an integrated approach is particularly important in protected cultivation systems, where repeated cropping can facilitate the persistence of viruses and their vectors and increase the risk of complex mixed infections.

The present study also highlights the practical value of HTS for agricultural disease surveillance, and the use of conventional RT-PCR to verify virus presence cannot be ruled out. For example, TMV and ShCV-related virus were annotated but could not be detected by RT-PCR, presumably due to assembly errors. However, HTS remains limited due to the limitations of the GenBank database; the possibility of unknown viruses still existing in the field cannot be ruled out.

The discovery of CuCV and CTyV from symptomatic cucumber plants allowed virus-specific primers to be developed and subsequently used to determine their occurrence in additional field samples. This discovery-to-detection workflow provides a practical strategy for incorporating emerging viruses into routine crop health monitoring. HTS-based surveillance can therefore complement conventional diagnostic methods by providing an early-warning system for newly emerging pathogens and supporting the development of targeted disease-management measures.

Overall, this study reveals a diverse and dynamic virus community associated with cucumber production in Taiwan and identifies CuCV and CTyV as emerging viruses requiring further attention. The high frequency of mixed infections and the substantial prevalence of several viruses emphasize that cucumber disease management should move beyond single-pathogen diagnosis toward integrated, surveillance-based approaches. Incorporating HTS for virus discovery and periodic monitoring, followed by rapid virus-specific molecular diagnostics, could improve early detection and support more effective integrated disease management in protected cucumber production systems.

## Figures and Tables

**Table 1:** Statistics of Illumina-sequenced read counts.

Min. Length	Max. Length	Mean Length	N50	N90	Number of Contigs	Total Number of Bases
251	18,909	914	1708	341	73,518	67,202,424

**Table 2:** BLAST alignment and annotation results against viruses.

Contig ID	Contig Length	Reads Number	Mean Depth	Best Fit nt Acc. No.	Reference Virus	Genus	Family	Length of Reference Sequence	nt Map Range	nt Identity (%)	Coverage (%)
c46648_g1	3330	375,971	16,927.1	ON013893	Cucumber mosaic virus (CMV) DSMZ PV-0453, RNA1	*Cucumovirus*	*Bromoviridae*	3359	18–3355	98.4	99.4
c46648_g4	3028	254,490	12,600.0	ON013894	Cucumber mosaic virus (CMV) DSMZ PV-0453, RNA2			3044	17–3040	98.4	99.3
c46648_g2	2194	346,993	23,712.7	ON013895	Cucumber mosaic virus (CMV) DSMZ PV-0453, RNA3			2216	19–2212	98.4	99.0
c47406_g1	6138	1,537,414	37,533.6	AB061774	Melon yellow spot virus (MYSV) JP, L RNA	*Orthotospovirus*	*Tospoviridae*	8918	4213–8917	98.6	52.8
c47410_g1	5863	1,143,531	29,219.0	MF469043	Melon yellow spot virus (MYSV) Physalis, M RNA			4904	2–3469	94.4	70.7
c47410_g2	2331	469,647	30,181.8	MF469043	Melon yellow spot virus (MYSV) Physalis, M RNA			4904	3487–4904	97.4	28.9
c47358_g1	2959	14,132,723	71,550.6	FJ386391	Melon yellow spot virus (MYSV) TW, S RNA			3244	1–2022	97.5	62.3
c47055_g1	4210	83,129	2959.5	MW051788	Watermelon silver mottle virus (WSMoV) DSMZ PV-0283, L RNA	*Orthotospovirus*	*Tospoviridae*	8916	9–4218	98.4	47.2
c47408_g1	9519	1,463,162	23,040.4	MF469051	Watermelon silver mottle virus (WSMoV) 2015_001, L RNA			8917	322–8913	86.7	96.4
c36147_g1	1406	45,843	4879.2	MW051789	Watermelon silver mottle virus (WSMoV) DSMZ PV-0283, M RNA			4872	4–2749	98.2	56.4
c36851_g1	2763	88,195	4781.0	MW051789	Watermelon silver mottle virus (WSMoV) DSMZ PV-0283, M RNA			4872	3410–4815	98.7	28.9
c47023_g2	3598	303,395	12,640.7	Z46419	Watermelon silver mottle virus (WSMoV), S RNA			3536	1–3536	97.9	100
c46859_g2	7867	2144	40.8	JQ904628	Cucurbit chlorotic yellows virus (CCYV) Beijing, RNA1	*Crinivirus*	*Closteroviridae*	8607	580–8445	99.9	91.4
c46884_g1	8024	4881	91.2	MH819191	Cucurbit chlorotic yellows virus (CCYV) SD, RNA2			8041	20–8039	99.9	99.7
c45922_g1	9118	6221	102.3	OP976048	Chieh-qua chlorotic virus [synonym: cucurbit chlorotic virus (CuCV)] CM, RNA1	*Crinivirus*	*Closteroviridae*	9157	29–9145	99.8	99.6
c44091_g1	7753	12,798	247.5	OP976049	CuCV CM, RNA2			7788	30–7776	99.8	99.5
c44091_g2	2305	447	29.0	PV404083	Sunn-hemp crinivirus (ShCV) LU, RNA1	*Crinivirus*	*Closteroviridae*	7539	69–210	89.4	6
c47255_g1	4358	1078	37.1	MW359100	Cucumber Bulgarian latent virus (CBLV) TW	*Tombusvirus*	*Tombusviridae*	4577	20–4378	99.6	95.2
c37816_g1	724	24	5.0	KX650855	Tobacco mosaic virus (TMV) Xch-Wh	*Tobamovirus*	*Virgaviridae*	5808	4748–5470	98.9	12.5
**Contig ID**	**Contig Length**	**Reads Number**	**Mean Depth**	**Best Fit aa Acc. No.**	**Reference Virus**	**Genus**	**Family**	**Length of Reference Sequence**	**aa Map Range**	**aa Identity (%)**	**Coverage (%)**
c40723_g1	1309	123	14.1	XHV14371	Blackberry virus X (BVX) MLW_151	Unclassified	Unclassified	1696	74–356	45.9	98
c43195_g2	2429	147	9.2	XHV14371	BVX MLW_151			1696	731–1305	33.7	70
c43195_g1	1648	105	9.5	XHV14371	BVX MLW_151			1696	1294–1681	53.6	90

**Table 3:** Comparison of virus detection rates and statistical significance in the same cucumber-producing area between 2022 and 2025.

Virus	2022 (*n* = 34)		2025 (*n* = 30)		Significance (*p*-Value) ^2^
	Positive No. (%)	95% CI ^1^	Positive No. (%)	95% CI	
CBLV	1 (2.9%)	0.5–14.9%	5 (16.7%)	7.3–33.6%	NS (0.0934)
CMV	3 (8.8%)	3.1–23.0%	0 (0.0%)	0.0–11.4%	NS (0.2424)
CCYV	4 (11.8%)	4.7–26.6%	25 (83.3%)	66.5–92.5%	**** (<0.0001)
CuCV	7 (20.6%)	10.4–36.8%	16 (53.3%)	36.1–69.8%	* (0.0115)
MYSV	34 (100%)	89.8–100.0%	27 (90.0%)	74.4–96.5%	NS (0.0988)
WSMoV	1 (2.9%)	0.5–14.9%	29 (96.7)	83.3–99.4%	**** (<0.0001)
CTyV	3 (8.8%)	3.1–23.0%	12 (40.0%)	24.6–57.7%	** (0.0064)
TMV	0 (0.0%)	0.0–10.4%	0 (0.0%)	0.0–11.6%	NS (>0.9999)

^1^ 95% Confidence Intervals (CI) were calculated using the Wilson score method. ^2^ Calculated by Fisher’s exact test comparing 2022 and 2025 data. * *p* < 0.05; ** *p* < 0.01, **** *p* < 0.0001; NS, not significant.

## Data Availability

All data generated and analyzed during this study are included in this published article.

## Supplementary Materials

The following supporting information can be downloaded at: https://www.mdpi.com/article/10.3390/v18101150/s1, Table S1: Primers used for viral genome sequencing; Table S2: Virus information used for sequence analysis of cucurbit chlorotic virus (CuCV); Table S3: Virus information used for sequence analysis of cucumber tymovirus (CTyV); Table S4: Primers used for virus detection; Table S5: Percentages of full-length (FL) genome and putatively encoded proteins of cucurbit chlorotic virus (CuCV) XZ-C-1 compared to other isolates.
