# Supplementary material for: High-Throughput Virome Surveillance Reveals Emerging Viral Threats to Cucumber Production in Taiwan
Source: Viruses. 2026 Oct 16;18(10):1150. doi: 10.3390/v18101150 (PMC13709489; doi:10.3390/v18101150)
Supplement: Supplementary file 1 [file viruses-18-01150-s001.zip › Table S1_Primers for viral genome sequencing_R1.pdf]

Table S1. Primers used for viral genome sequencing

Primer	Sequence	PCR size
CuCV		
CuCV-R1-5′end	ACATGGGAAATTTACTTTCCTTGTG	2158 bp
CuCV-R1-3R	CACAATTATCCTCATAAATTTCTG	
CuCV-R1-4F	CTATCATAATCCCACCAGAA	1868 bp
CuCV-R1-4R	ACCTGGGAAGCTTGGAGTCAG	
CuCV-R1-5F	CGCTTGATAGCATCCACGAT	2123 bp
CuCV-R1-5R	GCACTGATCCTTTCACCTATCTTGT	
CuCV-R1-6F	GAGATATAGAGTGATCACGGACAA	3230 bp
CuCV-R1-3′end	GACCTAGTTTATTGYTAACTAGTC	
CuCV-R2-5′end	ACATGGGGGAAATTTAATTTCCTTG	2621 bp
CuCV-R2-1R	CACCAGATCCATACTCCTTCATC	
CuCV-R2-2F	GATGAATTCGCTACTCTGATAGCA	2164 bp
CuCV-R2-2R	AAACGTGGTCTCTTAATGCT	
CuCV-R2-3F	GCAGCGCCGGTTTTCTATTAGT	3202 bp
CuCV-R2-3′end	GACCTAGTTTATTTATTAACTAAG	
CTyV		
c40723-1F	TGGGAAACTGACCCAAAGTC	981 bp
c40723-1R	CTCAACTTAGCTATTGCGCTTTC	
c43195-1F	GTATTGCCCTAGTTCCACTTCTTA	2253 bp
c43195-1R	CCACAGAAGCTCGGTCTCTTAC	
c40723-2F	ACCTTCGGAAACTTTGAGTGTCT	1866 bp
c43195-2R	GCCTAGAACCGTTTTAACACCTA	
c43195-3F	GCTGGAGATGACATGGCTTC	507 bp
c43195-3R	CCTGTGCAGCTACGTGTCTAG	
