# Supplementary material for: High-Throughput Virome Surveillance Reveals Emerging Viral Threats to Cucumber Production in Taiwan
Source: Viruses. 2026 Oct 16;18(10):1150. doi: 10.3390/v18101150 (PMC13709489; doi:10.3390/v18101150)
Supplement: Supplementary file 1 [file viruses-18-01150-s001.zip › Table S2_Virus accessions.pdf]

Table S2. Virus information for sequence analysis

Virus	Isolate	Host	Collection location	Accession number	
				RNA1	RNA2
Cucurbit chlorotic virus (CuCV)	XZ-C-1	*Cucumis sativus*	Taiwan	PQ618563	PQ618564
	KB	*Cucumis melo*	Thailand	MT813029	MT819949
	chieh-qua-CM	*Benincasa hispida* var. *chieh-qua*	China	OP976048	OP976049
	watermelon-LS	*Cucumis sativus*	China	OP976050	OP976051
	pumpkin-DF	*Cucurbita moschata*	China	OP976052	OP976053
	cucumber-SY	*Citrullus lanatus*	China	OP976054	OP976055
Cucurbit yellow stunting disorder virus (CYSDV)	AlLM	*Cucumis melo*	Spain	AY242077	AY242078
Cucurbit chlorotic yellows virus (CCYV)	TW	*Cucumis melo*	Taiwan	KY618798	KY618799
