# Supplementary material for: High-Throughput Virome Surveillance Reveals Emerging Viral Threats to Cucumber Production in Taiwan
Source: Viruses. 2026 Oct 16;18(10):1150. doi: 10.3390/v18101150 (PMC13709489; doi:10.3390/v18101150)
Supplement: Supplementary file 1 [file viruses-18-01150-s001.zip › Table S3_Tymovirales acc no.pdf]

Table S3. Virus information used for sequence analysis of cucumber tymovirus (CTyV)

Virus name	Abbreviation	Species	Genus	Family	Accession number
Shallot virus X	ShVX	*Allexivirus ecsascalonicum*	*Allexivirus*	*Alphaflexiviridae*	M97264
Botrytis virus X	BotVX	*Botrexvirus ecsbotrytis*	*Botrexvirus*		AY055762
Lolium latent virus	LoLV	*Lolavirus latenslolii*	*Lolavirus*		EU489641
Donkey orchid symptomless virus	DOSV	*Platypuvirus asinorchis*	*Platypuvirus*		KC923234
Sclerotinia sclerotiorum debilitation-associated RNA virus	SsDRV	*Sclerodarnavirus sclerotiniae*	*Sclerodarnavirus*		AY147260
Indian citrus ringspot virus	ICRSV	*Potexvirus citrindicum*	*Mandarivirus*		AF406744
Potato virus X	PVX	*Potexvirus ecspotati*	*Potexvirus*		M72416
Banana mild mosaic virus	BanMMV	*Banmivirus musae*	*Banmivirus*	*Betaflexiviridae*	AF314662
Apple stem grooving virus	ASGV	*Capillovirus mali*	*Capillovirus*		JN701424
Potato virus M	PotVM	*Carlavirus misolani*	*Carlavirus*		D14449
Angelica chordovirus	AChV	*Chordovirus angelicae*	*Chordovirus*		OR656535
Citrus leaf blotch virus	CLBV	*Citrivirus citri*	*Citrivirus*		OR766026
Diuris virus A	DVA	*Divavirus alphadiuris*	*Divavirus*		JX173276
Apple stem pitting virus	ASPV	*Foveavirus mali*	*Foveavirus*		D21829
Apricot vein clearing associated virus	AVCaV	*Prunevirus armeniacae*	*Prunevirus*		HG008921
Ribes americanum virus A	RAVA	*Ravavirus alpharibis*	*Ravavirus*		MF166685
Cherry rusty mottle associated virus	CRMaV	*Robigovirus robigomaculae*	*Robigovirus*		KC218926
Sugarcane striate mosaic associated virus	SCSMaV	*Sustrivirus sacchari*	*Sustrivirus*		AF315308
Cherry virus T	ChVT	*Tepovirus tafavii*	*Tepovirus*		PZ351581

Grapevine berry inner necrosis virus	GbINV	*Trichovirus necroacini*	*Trichovirus*		D88448
Grapevine virus A	GVA	*Vitivirus alphavitis*	*Vitivirus*		X75433
Watermelon virus A	WVA	*Wamavirus alphacitrulli*	*Wamavirus*		KY363796
Hobart betaflexivirus 1	HBV-1	Unclassified	Unclassified		MG995738
Grapevine Kizil Sapak virus	GKSV	Unclassified	Unclassified		MK204655
Citrus yellow spot virus	CYSV	Unclassified	Unclassified		MN879752
Hibiscus chlorotic speck associated virus 1	HCSaV-1	Unclassified	Unclassified		PP531524
Fusarium graminearum deltaflexivirus 1	FgDFV1	*Deltaflexivirus fusarii*	*Deltaflexivirus*	*Deltaflexiviridae*	KX015962
Sclerotinia sclerotiorum deltaflexivirus 1	SsDFV1	*Deltaflexivirus sclerotiniae*	*Deltaflexivirus*		KT581451
Botrytis virus F	BotVF	*Mycoflexivirus phibotrytidis*	*Mycoflexivirus*	*Gammaflexiviridae*	AF238884
Pistacia-associated flexivirus 1	PaFV1	*Gammaflexivirus unipistaciae*	*Gammaflexivirus*		MK605686
Entoleuca gammaflexivirus 2	EntGFV2	*Xylavirus duoentoleucae*	*Xylavirus*		MF375884
Grapevine fleck virus	GFkV	*Maculavirus vitis*	*Maculavirus*	*Tymoviridae*	AJ309022
Maize rayado fino virus	MRFV	*Marafivirus maydis*	*Marafivirus*		AF265566
Turnip yellow mosaic virus	TYMV	*Tymovirus brassicae*	*Tymovirus*		X07441
Blackberry virus X	BVX	Unclassified	Unclassified	Unclassified	PQ317258
