# Supplementary material for: High-Throughput Virome Surveillance Reveals Emerging Viral Threats to Cucumber Production in Taiwan
Source: Viruses. 2026 Oct 16;18(10):1150. doi: 10.3390/v18101150 (PMC13709489; doi:10.3390/v18101150)
Supplement: Supplementary file 1 [file viruses-18-01150-s001.zip › Table S4_Primers for virus detection_R1.pdf]

Table S4. Primers used for virus detection

Primer	Sequence	Target	PCR size	Reference
CBLV3900F	AAAGATTATCCCCTTGGTTTCA	CBLV MP	681 bp	[6]
CBLV4576R	GGGCTGCCTTTCGGCAATGTTC	CBLV MP		[6]
Crini-hsp70-f	GCCATAACCATTACGGGAGA	CCYV HSP70h	389 bp	[22]
Crini-hsp70-r	CGCAGTGAAAAACCCAAACT	CCYV HSP70h		[22]
CMV-CP-Nco-F	GCCATGGACAAATCCGAATCAACC	CMV CP	652 bp	Our laboratory, unpublished
CMV-CP-Sac-R	GGAGCTCCCCTGATGTGGGAAT	CMV CP		Our laboratory, unpublished
MY-N516f	GCTATGCATTGGAGGACCTCTATC	MYSV N	259 bp	[23]
MY-N774r	GGATATGCCACTGGTTGTTCCTGG	MYSV N		[23]
WS-N572f	TCTAGTATAAGGGGACTAGCACCGA	WSMoV N	267 bp	[24]
WS-N-808r	CCTGTCCATGTACTTAACCTGATCC	WSMoV N		[24]
TMVmpf	AGTTGTTGATGAGTTCATGGA	TMV MP	815 bp	[25]
TMV3nr	CAACCCTTCGATTTAAGTGGA	TMV 3′UTR		[25]
CuCV-R2-1F	GAGACAGAGTTTATCCCGACG	CuCV HSP70h	1528 bp	This study
CuCV-R2-1R	CACCAGATCCATACTCCTTCATC	CuCV HSP70h		This study
c40723-1F	TGGGAAACTGACCCAAAGTC	CTyV	981 bp	This study
c40723-1R	CTCAACTTAGCTATTGCGCTTTC	CTyV		This study
c43195-1F	GTATTGCCCTAGTTCCACTTCTTA	CTyV	2253 bp	This study
c43195-1R	CCACAGAAGCTCGGTCTCTTAC	CTyV		This study
c44091-1F	CATCACCTAAGTCTCCGAAAGG	ShCV-annotated	1328 bp	This study
c44091-1R	CCCTTGAAGTCACTGGAGATATG	ShCV-annotated		This study
