# Supplementary material for: High-Throughput Virome Surveillance Reveals Emerging Viral Threats to Cucumber Production in Taiwan
Source: Viruses. 2026 Oct 16;18(10):1150. doi: 10.3390/v18101150 (PMC13709489; doi:10.3390/v18101150)
Supplement: Supplementary file 1 [file viruses-18-01150-s001.zip › Table S5_CuCV genome comparison.pdf]

Table S5. Percentages of full-length (FL) genome and putatively encoded proteins of cucurbit chlorotic virus (CuCV) XZ-C-1 compared to other isolates

RNA1

Isolate	FL	1a		1b		p5		p25		p23	
	nt	nt	aa	nt	aa	nt	aa	nt	aa	nt	aa
pumpkin-DF	99.0	98.6	98.8	99.9	100.0	99.3	100.0	99.8	100.0	99.7	99.5
cucumber-SY	98.3	98.3	98.5	98.6	99.6	97.1	95.6	98.4	98.1	98.1	98.4
watermelon-LS	99.4	99.2	99.1	99.6	100.0	98.6	97.8	99.8	100.0	99.7	99.5
chieh-qua-CM	99.8	99.7	99.8	99.9	100.0	100.0	100.0	99.8	100.0	99.3	99.0
KB	99.2	99.2	99.6	99.5	99.4	99.3	97.8	98.9	98.6	98.8	99.0
CYSDV-AlLM	65.2	65.1	65.2	76.7	90.1	45.2	11.1	51.0	23.1	55.9	45.3
CCYV-TW	59.7	58.9	48.2	71.2	78.2	-	-	-	-	44.5	10.4

RNA2

Isolate	FL	p5		HSP70h		p7		p60		p10		CP		CPm		p27	
	nt	nt	aa	nt	aa	nt	aa	nt	aa	nt	aa	nt	aa	nt	aa	nt	aa
pumpkin-DF	99.8	98.3	94.7	99.7	99.8	100.0	100.0	99.9	99.6	99.6	98.7	100.0	100.0	99.9	99.8	99.4	98.7
cucumber-SY	99.7	98.3	94.7	99.6	100.0	100.0	100.0	99.7	99.8	99.6	98.7	99.7	100.0	99.6	99.4	100.0	100.0
watermelon-LS	99.7	97.4	92.1	99.8	100.0	100.0	100.0	100.0	100.0	99.6	98.7	99.7	100.0	99.8	99.4	99.1	98.3
chieh-qua-CM	99.9	98.3	94.7	99.7	100.0	100.0	100.0	100.0	100.0	99.6	98.7	99.9	100.0	99.9	99.8	100.0	100.0
KB	99.3	95.7	92.1	99.6	100.0	99.4	100.0	99.4	100.0	99.6	98.7	99.5	99.2	99.4	98.5	99.4	100.0
CYSDV-AlLM	66.8	47.0	5.9	74.8	83.9	60.6	50.0	68.3	70.8	59.6	54.4	70.8	74.1	63.6	56.3	69.3	71.5
CCYV-TW	60.1	46.5	5.7	69.5	73.8	58.2	50.0	59.5	55.5	57.7	39.2	66.2	63.4	55.9	39.5	54.4	41.3
